# Impact of nerve‐sparing posterolateral parametrial excision for deep infiltrating endometriosis on postoperative bowel, urinary, and sexual function

**DOI:** 10.1002/ijgo.14089

**Published:** 2022-01-20

**Authors:** Manuel Maria Ianieri, Diego Raimondo, Andrea Rosati, Laura Cocchi, Rita Trozzi, Manuela Maletta, Antonio Raffone, Federica Campolo, Giuliana Beneduce, Antonio Mollo, Paolo Casadio, Ivano Raimondo, Renato Seracchioli, Giovanni Scambia

**Affiliations:** ^1^ Division of Gynecological Oncology Department for the Protection of Women's and Children's Health and Public Health Fondazione Policlinico Universitario A. Gemelli IRCCS Università Cattolica del Sacro Cuore Rome Italy; ^2^ Division of Gynecology and Human Reproduction Physiopathology Department of Medical and Surgical Sciences (DIMEC) IRCCS Sant’Orsola‐Malpighi Hospital University of Bologna Bologna Italy; ^3^ Gynecology and Obstetrics Unit Department of Neuroscience, Reproductive Sciences and Dentistry School of Medicine University of Naples Federico II Naples Italy; ^4^ Gynecology and Obstetrics Unit Department of Medicine, Surgery and Dentistry "Schola Medica Salernitana" University of Salerno Baronissi Italy; ^5^ School in Biomedical Sciences University of Sassari Sassari Italy

**Keywords:** deep infiltrative endometriosis, functional outcomes, nerve‐sparing, parametrectomy, pelvic nerves

## Abstract

**Objective:**

To evaluate the functional outcomes of nerve‐sparing surgery for deep infiltrating endometriosis (DIE) with or without posterolateral parametrectomy.

**Methods:**

A multicenter, observational, retrospective, cohort study was performed including all symptomatic women who underwent nerve‐sparing laparoscopic excision of DIE and preoperative and postoperative assessment of functional outcomes through validated questionnaires between April 2019 and March 2020. Women with posterolateral parametrial DIE (P‐group) and women with no parametrial involvement (NP‐group) were compared in terms of preoperative and postoperative functional outcomes related to pelvic organs assessed through validated questionnaires (KESS and GIQLI for bowel function, BFLUTS for urinary function, and FSFI for sexual function); pain symptoms at 3‐month follow up assessed through an 11‐point visual analogue scale (VAS) for dyschezia, dysmenorrhea, dyspareunia and chronic pelvic pain; surgical outcomes; and rate of urinary voiding dysfunction at 3‐month follow up.

**Results:**

One‐hundred patients were included: 69 in the P‐group and 31 in the NP‐group. Preoperative and postoperative values of questionnaires, pain symptoms, and postoperative complication rates were comparable between the two groups, except for postoperative dyspareunia and sexual dysfunction, which were statistically higher in the P‐group. Only patients in the P‐group experienced urinary voiding dysfunction, but no statistical significance was reached (*P* = 0.173).

**Conclusion:**

Posterolateral parametrectomy for DIE appears to be associated with a higher risk of postoperative dyspareunia and sexual dysfunction.

## 1 INTRODUCTION

Endometriosis is defined as the presence of ectopic endometrial glands and stroma. Deep infiltrating endometriosis (DIE), defined as endometrial glands and stroma infiltrating the peritoneum by at least 5 mm, is the most severe form of endometriosis.^1^

Within the DIE spectrum, posterolateral parametrium is one of the most frequent localizations and is characterized by severe pain symptoms and pelvic dysfunction, reducing women's quality of life.^2^

Lateral and posterior parametria may be defined as areas of connective tissue extending from the uterus to the pelvic wall and enveloping vascular and autonomous nerve structures.^3^, ^4^ In particular, the lateral parametrium consists of connective mesenteries formed by areolar tissue enveloping visceral branches of the hypogastric vessels during their course towards the uterus and vagina.^2^ On the other hand, the posterior parametrium corresponds to the uterosacral ligament plus rectovaginal ligaments and lateral ligament of the rectum.^4^

Surgery for DIE, and in particular for posterolateral parametrium DIE, has been proved to be associated with severe iatrogenic pelvic organ dysfunctions, due to accidental injury of pelvic nerves.^5^ Over time, the principles of nerve‐sparing surgery have been incorporated into the surgical treatment for DIE in order to minimize such iatrogenic damages and potentially reduce the risk of functional complications.^6^, ^7^ Few cohort studies have investigated the functional outcomes of nerve‐sparing surgery for DIE with contrasting findings.^6^, ^8^, ^9^, ^10^, ^11^, ^12^

The aim of this study was to evaluate functional outcomes of nerve‐sparing surgery for DIE with or without posterolateral parametrectomy.

## 2 MATERIALS AND METHODS

### 2.1 Study protocol

The study was designed as a multicenter, observational, retrospective, cohort study and was reported according to the The Strengthening the Reporting of Observational Studies in Epidemiology (STROBE) guidelines and checklist.^13^

Electronic databases and clinical records were searched for symptomatic women who underwent nerve‐sparing laparoscopic excision of DIE and preoperative and postoperative assessment of functional outcomes through validated questionnaires and of pain (i.e. dyschezia, dysmenorrhea, dyspareunia, and chronic pelvic pain) between April 2019 and March 2020 at two tertiary academic centers.

Exclusion criteria were absence of fulfilled questionnaires for functional outcomes assessment; diagnosis of other medical or surgical conditions altering pelvic organ function (i.e. multiple sclerosis, irritable bowel syndrome); absence of sexual activity; gross involvement of hypogastric nerves or inferior pelvic plexus during surgical dissection; age <18 years.

Patients were divided into two groups according to the presence of posterolateral parametrial involvement (P‐group) or not (NP‐group) at surgery. Preoperative and postoperative functional outcomes, as well as differences between the preoperative and postoperative outcomes were compared among the groups.

### 2.2 Study outcomes

Primary outcome was the comparison in functional outcomes related to pelvic organs (bowel, urinary, and sexual function) assessed through validated questionnaires between the P and NP groups.

Secondary outcomes were the comparison between the P and NP groups in terms of:

- pain symptoms at 3‐month follow‐up evaluation;
- surgical outcomes, such as intraoperative and perioperative complications, rate of open conversion, additional surgical procedures, blood loss, time of hospitalization, and operating time;
- rate of urinary voiding dysfunction at 3‐month follow‐up evaluation.

In particular, the Gastrointestinal Quality of Life Index (GIQLI)^14^ and the Knowles‐Eccersley‐Scott‐Symptom Questionnaire (KESS)^15^ questionnaires were considered for assessing bowel function, the Bristol‐Female‐Lower‐Urinary‐Tract‐Symptoms (BFLUTS) questionnaire^16^ was used for urinary function, and The Female‐Sexual‐Function‐Index (FSFI)^17^ questionnaire was used for sexual function.

In detail, the GIQLI questionnaire describes health‐related quality of life related to gastrointestinal function and contains 36 questions with a total ranging from 0 to 144, which represents the best quality of life score.^14^ On the other hand, the KESS questionnaire assesses the bowel function with a specific focus on constipation,^15^ consists of 11 items evaluating different aspects of gastrointestinal function. The total KESS ranges from 0 to 39 points. A cut‐off of KESS score equal or superior to 10 points was used to define constipated patients.^15^

The BFLUTS questionnaire comprises 19 symptom questions investigating incontinence, voiding dysfunctions, and filling troubles. The sum provides a total score ranging from 0 to 45, where higher scores correspond to increased bladder dysfunction.^16^

The FSFI questionnaire is based on 19 questions exploring all domains of sexual function. The total score varies from 2 to 36, with higher scores indicating better overall sexual functioning. We considered a total FSFI score <26.5 suggestive for female sexual dysfunction.^17^

Regarding assessment of pain symptoms, 11‐point visual analogue scales (VAS) for dyschezia, dysmenorrhea, dyspareunia, and chronic pelvic pain were considered.

Urinary voiding dysfunction was defined as urinary retention greater than 100 ml after two attempts of post‐voiding urinary volume control with extemporary catheterization during postoperative hospitalization. Self‐bladder catheterization was recommended until the residual volume was <100 ml in three consecutive measurements.

### 2.3 Preoperative evaluation

At pre‐operative evaluation, all women underwent collection of medical and surgical history, rectovaginal examination, and transvaginal and transabdominal ultrasonography to map the endometriotic lesions. An interview about pain symptoms severity and questionnaires about urinary, gastrointestinal and sexual function were administered.

### 2.4 Surgical procedures

All patients were operated on by surgical teams with wide experience in laparoscopic surgical excision of DIE.

The severity of the disease was intraoperatively classified according to the revised American Society for Reproductive Medicine score.^18^

In all cases, the surgical approach for posterior DIE used a nerve‐sparing approach as previously published.^6^, ^7^, ^19^ When DIE involved the lateral and/or posterior parametrium, a nerve‐sparing parametrectomy was performed, as previously described.^2^ In particular, posterior parametrectomy was performed using interfascial dissection between parietal and visceral pelvic fasciae as described in a previous cadaveric and in vivo study.^7^ “Opening of the posterior parietal peritoneum at the level of the sacral promontory medial to the infundibulopelvic ligament of the ovary or at the level of the Douglas’ pouch; caudal extension of the peritoneal incision for 3–5 cm toward the juxtacervical insertion of the uterosacral ligament. Medialization of the rectum and partial development of the medial pararectal space paying attention to identify and preserve the ‘hypogastric’ fascia enveloping hypogastric nerves, following the cleavage plane between it and the rectal wall enveloped by the fascia propria recti. When needed, a peritoneal incision at the level of the rectouterine pouch allows the development of rectovaginal space and identification of the posterolateral parametrium with conservation of the nerve structures into rectal wings and ‘hypogastric’ fascia. If grossly involved by the disease, rectal wings and/or rectovaginal ligaments and/or uterosacral ligaments were resected. Regarding lateral parametrectomy, after development of lateral pararectal spaces, the uterine artery was isolated from its origin to ureteral tunnel, and uncrossing between the ureter and uterine artery was performed”.^2^, ^7^ When possible, the uterine artery was spared, and the deep uterine vein was used as a landmark to distinguish the vascular portion of the paracervix from its neural portion, which was preserved in case of deep parametrial resection.^2^, ^7^

In the case of recto‐sigmoid endometriosis, a shaving technique was attempted first. If residual nodule was present, then segmental resection or discoid resection was performed according to the longitudinal diameter, distance from anus, and circumferential involvement of bowel lesions.^20^

When ureteral involvement by the disease was intraoperatively observed, ureterolysis was performed first; if this latter failed to solve ureteral infiltration, ureteral resection was executed with end‐to‐end anastomosis or reimplantation.^21^

### 2.5 Postoperative data

Postoperative complications, occurring within 30 days after surgery, were registered and described according to Clavien‐Dindo classification.^22^ Three months from surgery, patients underwent rectovaginal evaluation, and transvaginal and transabdominal ultrasonography. Interviews on pain symptoms and questionnaires were also carried out at this time.

### 2.6 Statistical analysis

Statistical analyses were performed using SPSS version 27.0 (IBM, Armonk, NY). Qualitative variables were described with percentages, while quantitative ones were summarized using mean ± standard deviation or median and range. Comparisons between categorical variables were performed with chi‐squared test or Fisher exact test when required. Comparisons between continuous variables have been performed with Student's *t* test or Wilcoxon‐Mann‐Whitney test when appropriate. A two‐way mixed model analysis of variance for repeated measures was applied for comparison where the within‐subjects factor is “time” (two measurements) and the between‐subjects factor is “group” (parametrectomy vs no parametrectomy). A *P* value <0.05 was considered significant.

### 2.7 Ethical statement

The study received approval from the Institutional Review Board (CE‐AVEC 978/2020/Oss/AOUBo approved, November 18, 2020; CE‐Policlinico Gemelli protocol number 0029281/20, July 13, 2020) and was carried out according to the Helsinki Declaration. During preoperative evaluation, patients were asked to sign in advance a consent to the subsequent use of their anonymized data.

## 3 RESULTS

### 3.1 Study population

During the study period, 106 patients underwent surgery for DIE, six were excluded for a gross infiltration of the hypogastric nerve or the inferior pelvic plexus. One hundred patients met the inclusion criteria and were considered for the study analyses.

Clinical and demographic characteristics of the study groups are described in Table 1. Age and previous surgery were similar between the two groups. The mean body mass index (calculated as weight in kilograms divided by the square of height in meters) was statistically higher in the NP‐group (22 [20–24.8] in the P‐group vs 25 [22.8–29] in the NP‐group, *P* = 0.002). No significant difference was revealed between the proportion of patients with previous surgery for endometriosis or distribution of revised American Society for Reproductive Medicine stages among the two groups.

**TABLE 1:** Clinical and demographic characteristics of parametrectomy group (P) and non‐parametrectomy group (NP)^a^

	Group P (*n* = 69)	Group NP (*n* = 31)	*P* value
Age, years	38 (32.5–43)	38 (34–46)	0.305
BMI	22 (20–24.8)	25 (22.8–29)	0.002
Previous surgery for endometriosis	19 (27.5)	14 (45.2)	0.083
Stage of disease accordingto rASRM classification			
IV	37 (53.6)	16 (51.6)	0.598
III	30 (43.5)	15 (48.4)	
II	2 (2.9)	0 (0)	

Abbreviations: BMI, body mass index (calculated as weight in kilograms divided by the square of height in meters); r‐ASRM, revised American Society for Reproductive Medicine.

^a^ Values are presented as median (range) or as number (percentage).

### 3.2 Perioperative data

Descriptions of intraoperative variables are summarized in Table 2. No laparotomic conversions or intraoperative complications were observed. Histopathologic examination confirmed DIE in all cases. Parametrectomy was performed in 69 (69%) patients; but was not necessary in the remaining 31 (31%) patients. Among the P‐group, 43 (62.3%) patients required unilateral parametrial resection, whereas the remaining 26 (37.7%) required a bilateral one. Regarding the extension, 29 (42%) patients underwent only posterior parametrectomy, whereas 40 (58%) needed also lateral parametrial excision. Additional surgical procedures were comparable between the two groups. Median blood loss and hospital stay were similar in both groups, but the operative time was longer in the P‐group than the NP‐group (*P* = 0.047).

**TABLE 2:** Surgical details of parametrectomy group (P) and non‐parametrectomy group (NP)^a^

	Group P (*n* = 69)	Group NP (*n* = 31)	*P* value
Bowel surgery	45 (65.2)	23 (74.2)	0.373
Rectal shaving	21 (30.4)	10 (32.3)	0.855
Segmental bowel resection	18 (26.1)	8 (25.8)	0.976
Discoid resection	10 (14.5)	6 (19.4)	0.540
Type of bowel anastomosis			
Termino‐terminal	5 (7.6)	4 (15.4)	0.513
Latero‐terminal	9 (13.6)	5 (19.2)	
Latero‐lateral	1(1.5)	0 (0)	
Hysterectomy	16 (23.2)	8 (25.8)	0.777
Ileostomy	5 (7.2)	1 (3.2)	0.434
Endometrioma stripping	33 (47.8)	12 (38.7)	0.397
Ureterolysis	58 (84.1)	22 (71)	0.130
Ureteral resection and reimplantation	3 (4.3)	0 (0)	0.550
Partial resection of the bladder	5 (7.2)	2 (6.5)	0.999
Excision of vaginal nodule	11 (15.9)	4 (12.9)	0.694
Estimated blood loss, ml	120 (100–150)	120 (120–150)	0.294
Operative time, min	180 (127–250)	154 (130–195)	0.047

^a^ Values are presented as median (range) or as number (percentage).

Descriptions of postoperative data are summarized in Table 3. The rate of postoperative complications was homogeneous between the two groups. We reported three (3%) grade 3 postoperative complications. Two patients in the NP‐group experienced bowel leakage; of them, one patient underwent Hartmann's procedure, while the second one (with a primary protective ileostomy) was treated conservatively with antimicrobial therapy and percutaneous intraperitoneal drainage. Lastly, one patient in the P‐group experienced hemoperitoneum requiring re‐intervention.

**TABLE 3:** Postoperative variables of parametrectomy group (P) and non‐parametrectomy group (NP)^a^

	Group P (*n* = 69)	Group NP (*n* = 31)	*P* value
Days of hospitalization	6 (4–8)	7 (4–8)	0.378
Fever^b^	6 (8.7)	3 (9.7)	0.999
Blood transfusion^b^	5 (7.2)	1 (3.2)	0.663
Hemoperitoneum^b^	1 (1.4)	0 (0)	0.999
Urinary tract infection^b^	4 (5.8)	1 (3.2)	0.999
Urinary voiding dysfunction^b^	6 (8.7)	0 (0)	0.173
Anastomotic leakage^b^	0 (0)	2 (6.5)	0.094
Complications according to Clavien‐Dindo classification^c^			
Grade 1	4 (5.8)	0 (0)	0.205
Grade 2	14 (20.3)	3 (9.7)	
Grade 3	1 (1.4)	2 (6.5)	

^a^ Values are presented as median (range) or as number (percentage).

^b^ Calculate considering the absolute numbers of each complication.

^c^ Calculate considering only the maximum Clavien‐Dindo grade for each patient.

### 3.3 Functional data and pain symptoms

Preoperative and postoperative scores of the questionnaires and pain symptoms were comparable between the two groups, except for postoperative female sexual dysfunction and dyspareunia, which were statistically higher in the P‐group (Table 4). The rate of constipated patients according to total KESS was comparable between the two groups, both before and after surgery.

**TABLE 4:** Functional data and pain symptoms at preoperative evaluation and 3‐month follow up of parametrectomy group (P) and non‐parametrectomy group (NP)

	Preoperative			Postoperative		
	Group P (*n* = 69)	Group NP (*n* = 31)	*P* value	Group P (*n* = 69)	Group NP (*n* = 31)	*P* value
Dyschezia VAS	0 (0–10)	3 (0–10)	0.194	0 (0–9)	0 (0–10)	0.783
Dysmenorrhea VAS	8 (0–10)	7 (0–10)	0.230	0 (0–10)	0 (0–8)	0.228
Dysuria VAS	0 (0–9)	0 (0–7)	0.224	0 (0–8)	0 (0–1)	0.415
Dyspareunia VAS	6 (0–10)	3 (0–10)	0.055	0 (0–8)	0 (0–3)	0.049
Chronic pelvic pain VAS	5 (0–10)	3 (0–10)	0.595	0 (0–8)	0 (0–8)	0.316
KESS total score	15 (3–29)	14 (2–25)	0.609	11 (0–28)	10 (0–31)	0.861
GIQLI total score	87 (42–129)	90 (8–105)	0.849	104 (59–139)	105 (60–138)	0.602
Constipation (KESS ≥10)	48 (69.6)	24 (77.4)	0.419	41 (59.4)	19 (61.3)	0.860
FSFI total score	21.6 (2–34)	23.7 (14–31)	0.101	24.4 (2–36)	26.1 (2–31)	0.397
BFLUTS total score	7 (0–30)	3 (1–58)	0.093	6 (0–34)	3 (1–36)	0.143
FSD (FSFI score <26.5)	45 (65.2)	20 (64.5)	0.946	39 (56.5)	10 (32.3)	0.025

Abbreviations: BFLUTS, the Bristol Female Lower Urinary Tract Symptom Questionnaire; FSD, female sexual dysfunction; FSFI, the Female Sexual Function Index; GIQLI, the Gastrointestinal Quality of Life Index; KESS, Knowles‐Eccersley‐Scott‐Symptom Questionnaire; VAS, visual analog scale.

^a^ Values are presented as median (range; expressed as minimum‐maximum) or as number (percentage).

Table 5 shows the results from the analysis of variance two‐way mixed model for repeated measures. For each variable of interest, there was a significant main effect of time, except for FSFI. Concerning the main effect of group, only dyspareunia presented a significant difference between the two groups (*P* = 0.015).

**TABLE 5:** Analysis of functional outcomes and pain symptoms with the two way mixed model analysis of variance for repeated measures^a^

	Group P (*n* = 69)		Group NP (*n* = 31)		Time main effect	Group main effect
	Preoperative	Postoperative	Preoperative	Postoperative		
Dyschezia VAS	3.2 ± 3.6	0.8 ± 2.0	4.2 ± 3.5	1.0 ± 2.5	<0.001	0.203
Dysmenorrhea VAS	6.5 ± 3.6	1.3 ± 2.6	5.5 ± 3.8	0.7 ± 1.8	<0.001	0.124
Dysuria VAS	1.2 ± 2.6	0.4 ± 1.4	0.5 ± 1.7	0.0 ± 0.2	0.026	0.079
Dyspareunia VAS	5.2 ± 3.6	0.9 ± 2.2	3.7 ± 3.5	0.1 ± 0.5	<0.001	0.015
Chronic pelvic pain VAS	4.1 ± 3.7	1.0 ± 2.1	3.5 ± 3.9	0.6 ± 1.9	<0.001	0.358
KESS total score	14.4 ± 6.3	11.8 ± 6.2	13.6 ± 6.3	12.0 ± 7.9	0.014	0.778
GIQLI total score	85.4 ± 19.2	101.5 ± 18.5	82.9 ± 20.0	102.5 ± 19.8	<0.001	0.838
FSFI total score	19.4 ± 9.8	21.6 ± 10.8	23.8 ± 3.7	23.7 ± 8.1	0.354	0.083
BFLUTS total score	9.1 ± 7.7	6.8 ± 5.6	8.3 ± 11.7	6.8 ± 7.6	0.006	0.788

Abbreviations: BFLUTS, the Bristol Female Lower Urinary Tract Symptom Questionnaire; FSFI, the Female Sexual Function Index; GIQLI, the Gastrointestinal Quality of Life Index; KESS, Knowles‐Eccersley‐Scott‐Symptom Questionnaire; NP, non‐parametrectomy group; P, parametrectomy group; VAS, visual analog scale.

^a^ Values are presented as mean ± standard deviation.

Six (8.7%) patients experienced urinary voiding dysfunction. Only one patient required self‐urinary catheterization up to the 3‐month follow‐up evaluation; among the remaining transitory urinary dysfunctions, the median time to complete restoration of voiding function was 45 days (range 20–60 days). Although without statistically significant difference (*P* = 0.173), all six patients (8.7%) with positive post‐voiding residual were in the P‐group.

## 4 DISCUSSION

This study shows that, although performed using a nerve‐sparing approach, the excision of posterolateral parametrial endometriosis seems to be associated with a higher risk of postoperative dyspareunia and sexual dysfunction compared with women without involvement of the parametria by DIE. On the other hand, there is no difference between the two groups in terms of urinary and bowel function nor in pain symptom control.

Parametria can be considered the "neurological electrical unit" of the pelvic viscera. Pelvic splanchnic nerves originate from the sacral roots S2–S3–S4, lie on the lateral aspect of the uterosacral ligament, and join with the hypogastric nerves, thus forming the pelvic plexus.^4^ The preservation of these fibers may avoid bladder and rectal dysfunctions and prevent reduction in vaginal lubrification and arousal.^6^, ^19^

Postoperative bladder voiding dysfunctions with need for self‐catheterization are some of the most frequent complications in DIE surgery, especially in cases of parametrial endometriosis.^6^, ^8^, ^9^, ^10^, ^22^, ^23^ Although without statistical significance in the comparison with the other group, in our study, 8.7% of patients with posterolateral parametrectomy experienced urinary voiding dysfunction, although only one of them required persistent self‐catheterization at 3‐month follow up.

Ballester et al.^9^ studied the impact of extraserosal pelvic fascia (EPF) resection on postoperative outcomes in patients with colorectal endometriosis. Also, with nerve‐sparing surgery, patients undergoing EPF resection more frequently required self‐catheterization (36.6% vs 22.6% during immediate postoperative course) and longer bladder catheterization (12.5% vs 7.8% over 3‐month follow up) than patients without EPF resection.

Assessing 34 women who underwent nerve‐sparing excision of posterior DIE with a certain degree of lateral compartment infiltration by the disease, Uccella et al.^12^ observed four cases (11.8%) of urinary voiding dysfunction immediately after surgery, with spontaneous improvement over time in all of them.

In a before and after study, Soares et al.^24^ found that self‐catheterization was required only in the retrospective cohort who did not undergo nerve‐sparing eradication of cul de sac endometriosis without specifying parametrial involvement by DIE.

Our study showed a significant improvement of urinary function after surgery, at the 3‐month follow‐up visit assessed thorough BFLUTS questionnaire, without difference between the P and NP groups. By using the same questionnaire before and after colorectal resection for endometriosis, Ballester et al.^25^ did not detect an impact of parametrial resection of the nerve‐sparing approach on BFLUTS scores at 1‐month follow up. However, different study population and follow‐up time can explain these discrepancies. Indeed, postoperative urinary dysfunction tends to improve over time.^23^

Regarding bowel function, our data showed a significant improvement over time in KESS (*P* = 0.014) and GIQLI (*P* < 0.001) after surgery for DIE, independent of the need for parametrial endometriosis excision. These data are in contrast with other studies,^26^, ^27^ in which, after segmental bowel resection for DIE, the authors did not observe relief from digestive complaints. In a recent study on discoid resection for bowel DIE, also D'Avout‐Fourdinier et al.^28^ reported a significant improvement of gastrointestinal function only in the GIQLI, but not in all validated questionnaires used before and after surgery (KESS, Wexner score, and Bristol scale). These differences in terms of improvement of gastrointestinal function could depend on the lower incidence of bowel endometriosis in our series.

The nerve‐sparing technique does not seem to improve gastrointestinal function after surgery for DIE, or in the case of bowel endometriosis. Spagnolo et al.^29^ did not find differences between preoperative and postoperative motility and sensory capacity of anorectal manometry, after nerve‐sparing surgery for a posterior DIE nodule greater than 30 mm. In addition, Ceccaroni et al.^6^ reported a non‐significant difference rate of constipation, and fecal and air incontinence after segmental bowel and parametrial resection for DIE in the group of patients undergoing nerve‐sparing surgery compared with the conventional approach.

Although surgery was associated with an improvement of dyspareunia in both groups, as already reported by several authors^6^, ^30^; in our study, nerve‐sparing posterolateral parametrectomy was associated with postoperative female sexual dysfunction. This may depend on damage to the autonomic nerves responsible of decreasing blood flow to the vagina and lubrification. In fact, the parasympathetic and sympathetic fibers represent the efferent arm of the spinal reflexes involved in neuroregulation of the female sexual response. These results agree with previous data reporting a reduction of sexual pleasure in 18% and 52% of patients undergoing nerve‐sparing parametrectomy and segmental bowel resection, respectively.^6^ On the other hand, Uccella et al.^12^ showed a significant improvement of FSFI after surgery for DIE; however, the results are not comparable because of the lack of a sub‐analysis for patients undergoing parametrectomy.^12^ A similar bias is found in other studies in which a general improvement is reported of dyspareunia^30^ or FSFI using or not a nerve‐sparing approach for DIE,^31^ without mention of the role of parametrectomy.

In accordance with the “interfascial approach”,^7^, ^19^ the preservation of the hypogastric fascia and the development of the space between it and the fascia propria recti allowed us to leave the neural structure beneath it virtually intact. However, in some cases, the resection of the disease requires a dissection caudal and lateral to the hypogastric fascia, leading to potential nerve injury. In these cases, different macroscopic and microscopic distributions of autonomic nerves, together with the neurotrophic properties of DIE lesions, make it difficult to perform a dissection nerve by nerve, which technically limits a nerve‐sparing approach.^7^, ^19^

The limits of the present study are a result of its retrospective design and tertiary level setting. Furthermore, a longer follow up might be needed to better evaluate any change of functional outcomes and pain control and anatomical recurrence of the disease.

Although performed using a nerve‐sparing approach, posterolateral parametrectomy for DIE seems to be associated with a higher incidence of dyspareunia and postoperative sexual dysfunction. Careful counseling about these associated risks must be performed before dealing with this complex surgery in order to provide the patient with informed choice.

## CONFLICTS OF INTEREST

The authors declare that they have no conflicts of interest and nothing to disclose.

## AUTHOR CONTRIBUTIONS

MMI and DR were responsible for study conception and design, data acquisition, statistical analysis, and manuscript drafting. AR, LC, RT, MM, and ARa contributed to study design, statistical analysis and interpretation, and manuscript drafting. FC and GB were responsible for data acquisition and contributed to manuscript drafting. AM was responsible for data analysis and interpretation and manuscript revision. PC, IR, RS, and GS were responsible for study conception and manuscript drafting and revision. All authors read and approved the final manuscript, and agreed to be accountable for all aspects of the work in ensuring that questions related to the accuracy or integrity of any part of the work are appropriately investigated and resolved.

## Data Availability

The data that support the findings of this study are available from the corresponding author upon reasonable request.
